# Bisorption of chromium(VI) from aqueous solutions by *Sargassum thunbergii* Kuntze

**DOI:** 10.1080/13102818.2014.907028

**Published:** 2014-07-03

**Authors:** Yun Wang, Yingxia Li, Feng Juan Zhao

**Affiliations:** ^a^ School of Life Science and Technology, Nanyang Normal University, Nanyang, P.R. China; ^b^ Marine Science and Engineering College, Qingdao Agricultural University, Qingdao, P.R. China; ^c^ School of Biological Sciences, BingZhou University, Binzhou, P.R.China

**Keywords:** chromium(VI), *Sargassum thunbergii*, Fourier transform infrared spectroscopy

## Abstract

The potential to remove Cr(VI) from aqueous solutions through biosorption using dried *Sargassum thunbergii* Kuntze was investigated with respect to initial pH, amount of biosorbent, initial Cr(VI) concentrations and temperature. Cr(VI) removal efficiency was influenced significantly by the variation of pH, and the optimum pH was about 2.0. Moreover, the biosorption kinetics followed the pseudo-second-order model. The adsorption equilibrium was well described by the Langmuir and Freundlich isotherm models. The maximum adsorption capacity was 1.855 mmol/g at 318 K and pH 2.0. The adsorption processes were endothermic and the biosorption heat was 15.8 kJ/mol. Furthermore, the Fourier transform infrared spectroscopy suggested that amido-, hydroxyl-, C = O and C—O groups were involved in the biosorption of Cr(VI) onto *S. thunbergii*.

## Introduction

Water pollution due to toxic heavy metals released by industrial activities is a serious environmental and public health issue because they tend to remain indefinitely circulating and eventually accumulating throughout the food chain.[1,2] Chromium is one of the key contaminants in the industrial wastewaters from different industries, e.g. dyes and pigments, film and photography, galavanometry and electrical equipment manufacture, leather and mining, plating and electroplating and metal-cleaning industries.[3] Cr(VI) is one of the most toxic heavy metals with its mutagenic and carcinogenic properties.[4] Hence, the removal of Cr(VI) from industrial drainage has become important to maintain water quality.

Various conventional processes, such as chemical precipitation, membrane filtration, ion exchange, reverse osmosis, evaporation and electrolysis, are usually applied to the treatment of industrial drainage. However, the application of such processes is often limited because of technical or economic constraints.[5] The main disadvantages are the high cost of implantation and operations for concentrations below 100 mg/L.[6] Therefore, new technologies with acceptable costs are necessary for reduction of the heavy metal concentration in industrial drainage.

Adsorption using low-cost biosorbents has been shown to be a feasible alternative technology involved in the removal of toxic metals from industrial drainage.[7] Adsorption of Cr(VI) from aquatic systems by using microbial biomass, including algae, fungi and bacteria, has gained importance. Marine algae with large quantities in many countries and high metal binding capacities are a promising biological adsorbent.[8] An earlier biosorption study was focused on unicellular and other microalgae [9] because of the large surface area for binding of metal ions. However, the high cost of harvesting them from cultivation or natural sources is a key hurdle in their industrial application. That is why, macroalgae are better suited for the purpose than microalgae.[10] Non-living seaweed biomass, particularly brown seaweed, has been found to be an excellent biosorbent for Cr(VI) due to its high adsorption capacity and the abundance of the biomass in many parts of the world's oceans.[11]

*Sargassum thunbergii* Kuntze is a brown macroalga species inhabiting the coastal areas of China, and widely distributed in warm and temperate water environments from northern Liaodong Peninsula to southern Leizhou Peninsula.[12] The present study investigates the potential use of untreated *S. thunbergii* biomass as a metal sorbent for removal of Cr(VI) from aqueous solution. This alga was chosen as adsorbent because of the relative lack of information about its sorption ability and abundance of the biomass in China. Environmental parameters affecting the biosorption process, such as pH, temperature, contact time, metal concentration and adsorbent concentration, were evaluated. The equilibrium adsorption data were evaluated by Langmuir, Freundlich, Redlich-Peterson and Temkin isotherm models. In addition, Fourier transform infrared spectroscopy (FTIR) was also employed to identify the specific functionalities involved in chromium binding to the seaweed.

## Materials and methods

### Algal biomass preparations

*S. thunbergii* was collected along the seaside of Qingdao, China. The algae were washed several times with distilled water to remove dirt and dried in an oven at 60 °C for 48 hours. The dried algae were fragmented and the particles with an average size of 0.5 mm were used for the experiments.

### Batch experiments

Each experiment was performed by bringing into contact 100 mL of a Cr(VI) solution of known concentration with the desired amount of biomass in a 250 mL flask. The stock chromium solution (100 mmol/L) was obtained by dissolving an exact quantity of K_2_Cr_2_O_7_ (GR, Aladdin Industrial Corporation, China) in distilled water. The working solutions of different concentrations were acquired by diluting the stock solution. The following parameters were chosen as the standard conditions: 1.0 g/L of biomass, 2 mmol/L of initial Cr(VI) concentration at pH 2.0 and 25 °C. To study the effect of biomass concentration on the Cr(VI) reduction rate, biomass concentrations of 0.4, 0.8, 1.2, 1.6, 2.0, 2.4, 2.8, 3.2, 3.6 and 4.0 g/L were employed. To study the effect of the initial Cr(VI) concentration, concentrations of 0.5, 1, 1.5, 2, 2.5, 3, 3.5, 4, 4.5 and 5 mmol/L were used. To study the effect of pH, the pH value of metal solutions was adjusted to 2.0, 3.0, 4.0, 5.0, 6.0, 7.0, 8.0 or 9.0 by employing different Cr concentration (1, 2 and 4 mmol/L) and 4 g/L biomass dosage. To study the effect of temperature, the flasks were incubated at 25, 35 or 45 °C. The flasks were agitated on a shaker at 1500 r/min. In all the experiments, the solution pH was maintained at the desired value using 0.1 mol/L HCl or 0.1 mol/L NaOH solutions.

### Equilibrium studies of biosorption

At a constant temperature, the extent of adsorption on a particular adsorbent depends on the concentration of a metal ion and algal dosage. During biosorption, equilibrium is established between the adsorbed metal ions on the cell surface and the unadsorbed metal ions in the solution. The amount of Cr(VI) uptake by *S. thunbergii* in each flask was determined using the mass balance equation [13]:(1) 
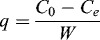
where *q* is the adsorption amount at equilibrium (mg/g), *C*_0_ is the concentration of heavy metal (mg/L), *C_e_* is the concentration remaining in solution at equilibrium (mg/L) and *W* is the biosorbent dosage (g/L).

### Analysis of chromium(VI) ions

The concentration of unadsorbed Cr(VI) ions in the biosorption medium was determined spectrophotometrically (UNICO UV 2000 recording spectrophotometer) at 540 nm, using diphenylcarbazide as the complexing agent.

### Fourier transform infrared analysis

The infrared spectra of the algae were obtained using a Fourier transform infrared spectrometer (NICOLET 5700, Thermo Electron Corporation). For the FTIR study, 30 mg of the biomass was encapsulated in 300 mg of KBr (Sigma) in order to prepare translucent sample disks.

## Results and discussion

### Effect of pH and adsorbent dose on Cr(VI) uptake

**Figure 1.**
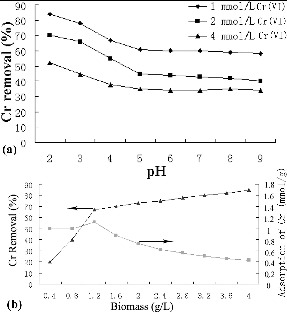
Cr(VI) removal efficiencies by *S. thunbergii* at different pH (a) and biomass (b). Initial Cr(VI) concentration in part (b) was 2 mmol/L; Cr removed percentage (▴); adsorption of Cr(VI) per unit weight of adsorbent (▪).

The initial pH of the metal solution is an important environmental factor affecting the biosorption of metal ions and affects not only the cell wall metal-binding sites, but also the metal chemistry in the water, e.g. hydrolysis, complexation by organic and/or inorganic ligands and redox reactions. Moreover, the precipitation, speciation and biosorption availability of the heavy metals are also strongly influenced by pH. In this study, Cr(VI) removal efficiency was influenced significantly by variation of pH (Figure 1(a)). The equilibrium experiments conducted at an initial pH varying from 2.0 to 9.0 under initial Cr(VI) concentrations of 1, 2 and 4 mmol/L showed that the maximum capacity was obtained at an initial pH 2.0. As the pH value increased, the removal percentage decreased gradually. The amount of Cr(VI) adsorbed from a 1 mmol/L solution at pH 2.0 was 84%, whereas a 43% reduction in biosorption was determined as the pH shifted from 2.0 to 5.0. The percentage of Cr ions adsorbed at pH 2.0 decreased with increasing Cr(VI) concentration. The adsorption of metal ions depends on the solution pH, which influences the electrostatic binding of ions to corresponding Cr groups. At the optimum sorption pH (pH 2.0), the dominant species of Cr ions in the solution are HCrO^4−^, Cr_2_O_7_^2−^, Cr_4_O_13_^2−^ and Cr_3_O_10_^2−^.[3] These Cr anions interact strongly with the negatively charged ions of the *S. thunbergii* matrix.

Another important factor affecting biosorption is the biomass concentration. The influence of adsorbent dosage on the percentage adsorption and equilibrium uptake is depicted in Figure1(b). The increase in adsorbent dosage from 0.4 to 1.2 g/L resulted in a pronounced increase in the adsorption percentage of Cr(VI) due to larger surface area and greater pore volume. Further increase in the adsorbent dosage (>1.2 g/L) did not cause significant improvement in adsorption. This might be attributed to the establishment of equilibrium between Cr(VI) ions bound to the sorbent and those remaining unadsorbed in the solution.[14]

However, Cr(VI) uptake showed a different trend to the percentage of adsorption (Figure 1(b)). With increase in the adsorbent dosage from 0.4 to 1.2 g/L, the adsorption of Cr(VI) per unit weight of adsorbent increased from 1 to 1.1 mmol/g. When the adsorbent dosage increased from 1.2 to 4 g/L, the adsorption of Cr(VI) per unit weight of adsorbent decreased from 1.1 to 0.43 mmol/g. Two factors might be responsible for this effect. First, the adsorption sites may remain unsaturated during the adsorption reaction at higher adsorbent concentrations. This could explain the relatively small increase in adsorption due to poorer utilization of the adsorptive capacity of the adsorbent with the dosage of adsorbent increasing. Second, the formation of cell aggregates at higher concentrations may lead to an increase in the diffusion path length and a decrease in the effective biosorption area. Particle interaction at higher adsorbent concentration might help to desorb some loosely bound Cr ions from the sorbent surface.[14]

### Kinetics of biosorption

**Figure 2.**
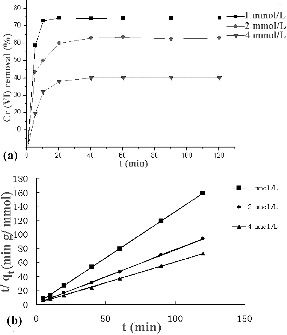
Adsorption rate (a) and plots of *t*/*q* vs. *t* (b) at different Cr(VI) concentrations.

The equilibrium time was dependent on the initial Cr(VI) concentration. The removal rate was rather fast in the first 10 minutes, and the respective equilibrium time under initial Cr(VI) concentrations of 1, 2 and 4 mmol/L was 20, 40 and 40 minutes, respectively (Figure 2(a)). After the first 10 minutes, slower non-linear adsorption was observed. In the initial 10 minutes, the Cr(VI) concentration was relatively high, and multiple ionic bonds formed on the algal cell surface. As the process continued, the Cr(VI) concentration decreased, which considerably reduced the rate of collision, and at the same time the number of available binding sites also decreased dramatically. Moreover, adsorbed Cr(VI) imparted hindrance to the neighbouring free binding sites. Thus, collective resistance to adsorption process increased manifold, which in turn slowed down the rate of adsorption.

The pseudo-second-order kinetic model based on the sorption capacity of solid phase was used in this case, assuming that the measured concentrations are equal to the cell surface concentrations.[8] The pseudo-second-order kinetic rate equations can be expressed as(2) 
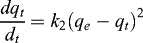



Integrating for the boundary conditions *q_t_* = 0, at *t* = 0, and *q_t_* at time *t*, the linearized form of the pseudo-second order model is obtained:(3) 
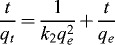
where *k*_2_ is the second-order biosorption rate constant, *q_e_* and *q_t_* are the amounts of sorbed metal ions on the biosorbent at the equilibrium and at any time *t*, respectively.

The plots of *t*/*q_t_* versus *t* at various initial concentrations are shown in Figure 2(b). The values of the parameters *k*_2_, *q_e_* and correlation coefficients are presented in Table 1. The correlation coefficients obtained were greater than 0.999, which suggested that the sorption of Cr(VI) onto *S. thunbergii* followed well the second-order model based on the assumption that the rate-limiting step might be chemisorption involving valence forces by exchange or sharing of electrons between sorbate and sorbent.[15]

**Table 1.** Pseudo-second-order adsorption constants.

Cr concentration (mmol/L)	*q_e_* (mmol/g)	*k*_2_ (g/mmol min)	*R*^2^
1	0.76	2.33	0.9999
2	1.30	0.44	0.9996
4	1.70	0.20	0.9990

### Adsorption isotherms

**Figure 3.**
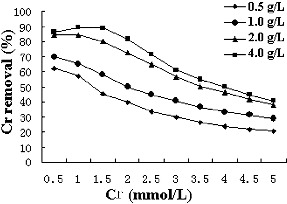
The effect of initial Cr(VI) concentrations (from 0.5 to 5 mmol/L) and different biomass concentrations (0.5, 1, 2 and 4 g/L) on the removal percentage.

The initial concentration of metal ions provides an important driving force to overcome all mass transfer resistances of metal ions between the aqueous and solid phases. The effect of different initial concentrations of Cr(VI) and different biomass concentrations on the removal of Cr(VI) via biosorption is shown in Figure 3. The adsorption percentage decreased the higher the initial Cr concentration was, corresponding to the metal ions on the biomass surface, which suggested that the surface saturation was also dependent on the initial metal ion concentration.

**Figure 4.**
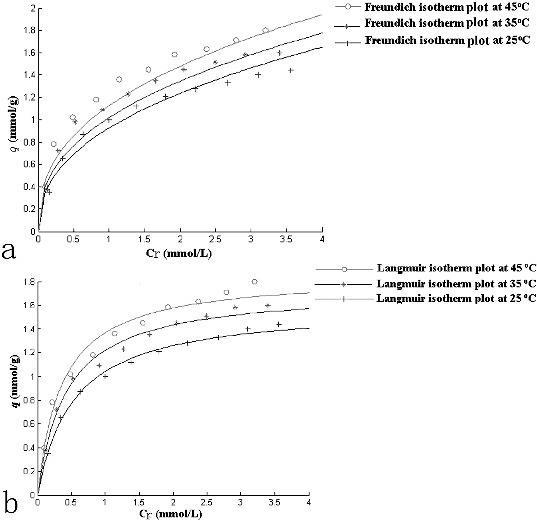
Freundlich (a) and Langmuir (b) isotherm plot at different temperatures.

Figure 4 indicated the non-linear relationship between the amount (mmol) of Cr(VI) sorbed per unit mass (g) of *S. thunbergii* against the concentration of Cr(VI) remaining in solution (mmol/L). Many methods were used for the adsorption studies, Freundlich and Langmuir adsorption isotherms were comprehensively adopted. Freundlich adsorption isotherm equation is empirical, while Langmuir adsorption isotherm equation is a classical expression.[16] The equilibrium relationship between the concentration of metal ions adsorbed per unit mass of original biosorbent (*q*_eq_ mmol/g) and the concentration of adsorbed metal ions at a constant temperature (*C*_eq_) is given by the adsorption isotherm. Out of many adsorption isotherms available, Freundlich and Langmuir adsorption isotherms were used to fit the data in the present study. The Freundlich adsorption isotherm is given as(4) 
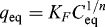

where *K_F_* and 1/*n* are Freundlich adsorption constants. The Langmuir isotherm is derived assuming a uniform surface with finite identical sites and monolayer adsorption of the adsorbate. The Langmuir adsorption isotherm is given by the relation:(5) 
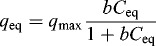
where *q*_max_ is the maximum adsorbate loading (mmol/L) and *b* is the Langmuir adsorption constant (mmol/L), which indicates the affinity of the sorbate for the solute.

The adsorption equilibria of Cr(VI) by *S. thunbergii* at different initial Cr(VI) concentrations were well described by the Langmuir and Freundlich isotherm models. The equilibrium constants and correlation coefficients (*R* ^2^) are presented in Table 2. The 1/*n* values were between 0 and 1, indicating that the biosorption of Cr(VI) using *S. thunbergii* biomass was favourable at the studied conditions. The maximum adsorption capacity of *S. thunbergii* obtained from the Langmuir isotherm was 1.855 mmol/g at 318 K.

**Table 2.** Langmuir and Freundlich sorption constants at different temperatures.

	Freundlich constants			Langmuir constants		
*T* (K)	*K_F_*	1/*n*	*R*^2^	*q_m_* (mmol/g)	*b* (L/mmol)	*R*^2^
298	0.925	0.417	0.954	1.596	1.884	0.999
308	1.018	0.4	0.949	1.736	2.358	0.996
318	1.126	0.392	0.951	1.855	2.809	0.991

### The biosorption heat of Cr(VI) onto *S. thunbergii*

The Langmuir model is based on a postulated physical or chemical interaction between solute and vacant sites on the adsorbent surface, and the heat (Δ*H*) of adsorption is independent of the fraction of surface covered by the adsorbed solute,(6) 
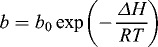
where *b*_0_ is a constant containing the entropy term, Δ*H* is the heat of the adsorption (kJ/mol), *R* is the universal gas constant (J/mol K^−1^) and *T* is the absolute temperature (K).

Equation (6) can be altered as(7) 
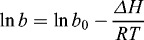



**Figure 5.**
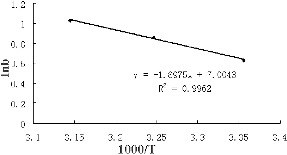
Heat of biosorption of Cr(VI) onto *S. thunbergii.*

The biosorption heat of Cr(VI) onto *S. thunbergii* was obtained by calculating the slope ln*b* versus 1/*T* (Figure 5). The negative slope value indicated the biosorption of Cr(VI) onto *S. thunbergii* to be endothermic. This was also supported by the increase of uptake capacity with the increasing temperature. The biosorption heat for Cr(VI) was 15.8 kJ/mol, which suggested that the adsorption of Cr(VI) to *S. thunbergii* was entropically governed.

### FTIR spectrum of *S. thunbergii*

**Figure 6.**
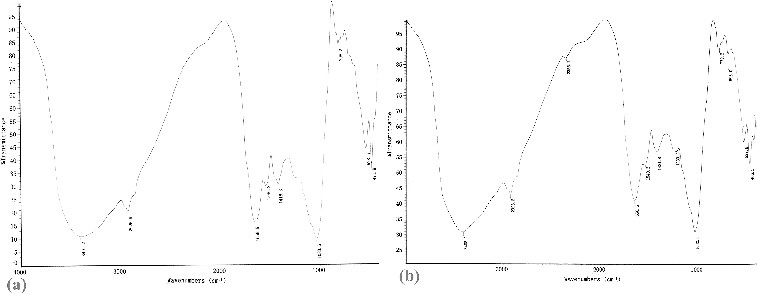
FTIR spectrum of unloaded (a) and Cr(VI)-loaded (b) *S. thunbergii.*

The absorbance spectrum of unloaded and Cr(VI) loaded *S. thunbergii* is shown in Figure 6. The broad and strong band at 3401.2 cm^−1^ was assigned to the stretching of hydrogen-bonded O—H, N—H of secondary amides and NH_3_^+^. The band peaks at 1656.5, 1540.2 and 1416.3 cm^−1^ were attributed to asymmetric and symmetric stretching vibration of C = O groups. The band at 1033.6 cm^−1^ was assigned to stretching of C—O groups on the biomass surface (Figure 6(a)). After Cr(VI) biosorption, the band at 3401.2 cm^−1^ was shifted to 3408.1 cm^−1^, and the bands observed at 1656.5 and 1416.3 cm^−1^ were shifted to 1650.6 and 1421.8 cm^−1^, respectively. The peak at 1033.6 cm^−1^ moved to 1032.3 cm^−1^ (Figure 6(b)). These results suggested that amido-, hydroxyl-, C = O and C—O groups could combine intensively with Cr(VI).

## Conclusions

The biomass of *S. thunbergii* has the potential to be used as an efficient and economic biosorbent for the removal and recovery of toxic Cr(VI) from wastewater due to high Cr(VI) biosorption and low cost. The operating parameters, pH of solution, biomass dosage, temperature and contact time, affected the biosorption capacity of *S. thunbergii* biomass. Kinetics fitted the pseudo-second-order kinetic model. The equilibrium data followed well the Langmuir and Freundlich isotherm models and the maximum adsorption capacity was 1.855 mmol/g at 318K and pH 2.0. The adsorption process was found to be endothermic and the biosorption heat was 15.8 kJ/mol. Amido-, hydroxyl-, C = O and C—O groups could be involved in the biosorption of Cr(VI) onto *S. thunbergii.*
